# Genome-wide analysis tracks the emergence of intraspecific polyploids in *Phragmites australis*

**DOI:** 10.1038/s44185-024-00060-8

**Published:** 2024-10-01

**Authors:** Cui Wang, Lele Liu, Meiqi Yin, Franziska Eller, Hans Brix, Tong Wang, Jarkko Salojärvi, Weihua Guo

**Affiliations:** 1 Institute of Ecology and Biodiversity, School of Life Sciences, Shandong University, Qingdao, China; 2 Shandong Provincial Engineering and Technology Research Center for Vegetation Ecology, Shandong University, Qingdao, China; 3 Organismal and Evolutionary Biology Research Program, Faculty of Biological and Environmental Sciences, University of Helsinki, Viikinkaari 1, Biocentre 3, Helsinki, Finland; 4 Department of Biology, Aarhus University, Aarhus, Denmark; 5 College of Landscape Architecture and Forestry, Qingdao Agricultural University, Qingdao, China; 6 School of Biological Sciences, Nanyang Technological University, Singapore, Singapore

**Keywords:** Evolution, Evolutionary genetics, Phylogenetics, Population genetics

## Abstract

Polyploidization plays an important role in plant speciation and adaptation. To address the role of polyploidization in grass diversification, we studied *Phragmites australis*, an invasive species with intraspecific variation in chromosome numbers ranging from 2n = 36 to 144. We utilized a combined analysis of ploidy estimation, phylogeny, population genetics and model simulations to investigate the evolution of *P. australis*. Using restriction site-associated DNA sequencing (RAD-seq), we conducted a genome-wide analysis of 88 individuals sourced from diverse populations worldwide, revealing the presence of six distinct intraspecific lineages with extensive genetic admixture. Each lineage was characterized by a specific ploidy level, predominantly tetraploid or octoploid, indicative of multiple independent polyploidization events. The population size of each lineage has declined moderately in history while remaining large, except for the North American native and the US Land types, which experienced constant population size contraction throughout their history. Our investigation did not identify direct association between polyploidization events and grass invasions. Nonetheless, we observed octoploid and hexaploid lineages at contact zones in Romania, Hungary, and South Africa, suggestively due to genomic conflicts arising from allotetraploid parental lineages.

## Introduction

Polyploidization in the form of allopolyploidization, autopolyploidization, or segmental polyploidization, has been associated with speciation and shaping genetic diversity in the plant kingdom^1–3^. Polyploids have been suggested to outperform their diploid progenitors in highly stressed abiotic environments^4^, and hence have an access to a wider range of habitats. Successful polyploids may be more disposed towards evolving into invasive species^5^ and drive diploid ancestors to extinction by recurrent hybridization followed by genomic recombination and duplications, as seen in the well-known *Spartina* species^6^. However, several empirical studies have challenged this view^7^, and in contrast Martin and Husband^8^ suggested that differing ploidy levels might facilitate the coexistence of diploid and tetraploid congeners. After the founding polyploid event (or Whole Genome Duplication, WGD), genomic redundancy is reduced through major genomic arrangements including shuffling and deletion of chromosome segments in a process known as “diploidization”. This allows the newly formed genome to function cohesively in the new species^9,10^. WGD events are particularly common among grasses^11^. For example, all cereals have undergone rho, sigma and tau WGD events. The grass family (Poaceae), which includes more than 10,000 species today, originated from a common ancestor with five chromosomes^12^. A series of genome duplication and subsequent genome fractionation events, involving chromosomal structural rearrangements and loss of redundancy, led to a common ancestral genome with 12 chromosomes in cereals. This genome structure is shared across the early diverging grass subfamilies Anomochlooideae, Pharoideae, and Puelioideae^12,13^. Hence, all extant Poaceae species are paleopolyploids, having undergone polyploidization millions of years ago^13^.

A recent polyploidization event can be directly inferred when closely related species exhibit different chromosome counts or genome sizes. This phenomenon is often observed in species within the same genus. However, different ploidy levels can also occur within the same species, as seen in *Dupontia fisheri*, *Betula pendula*, *Lygeum spartum*, *Cynodon dactylon,* and *Phragmites australis*^14–18^. One critical step in characterizing the extent of polyploidization is to assess the ploidy level directly by counting chromosome numbers of individual metaphases, or by quantifying the genome size in the germplasm using techniques such as flow cytometry and comparing it to the size of a known diploid representative. However, these procedures can be tedious and require appropriate facilities, which can be challenging. With the advancement of next-generation sequencing (NGS) technology, large quantities of NGS reads now provide a chance to infer the ploidy levels through alleles of the genetic markers. Tools such as ploidyNGS^19^, ConPADE^20^ and nQuire^21^ have been developed to infer ploidy levels by fitting a model to the allele frequencies from mapping against the reference genome or calculating the frequency of haplotypic contigs. Therefore, in silico methods can accurately predict ploidy levels for some organisms when flow cytometry is not available.

The common reed, *Phragmites australis* (Cav.) Trin. ex Steud., is a tenacious cosmopolitan wetland species which provides habitats for small animals and considered as an important part of ecosystems. Similar to many other grass species, the common reed has a basal chromosome count of x = 12. However, its euploid chromosome counts are versatile, ranging from 3x to 12x (3x, 4x, 6x, 7x, 8x, 10x, 11x and 12x), or even mixoploid^22,23^. Tetraploid and octoploid individuals are most common in nature^22,23^. Substantial efforts to investigate the speciation process in *Phragmites* have been made, yet the history of polyploidization and its association with genetic lineages within this genus remains unclear^24–28^. To date, seven species have been described within *Phragmites*: *P. australis*, *P. mauritianus* Kunth, *P. frutescens* H. Scholz, *P. dioica* Hackel ex Conert, *P. berlandieri* E. Fourn., *P. japonicus* Steudel and *P. vallatoria* (Plunk. ex L.) Veldk^26^. Most of these species are distributed regionally, with *P. australis* being the only cosmopolitan species^22,26,29^. Ploidy variation was found in several species, e.g., *P. vallatoria* has 2x, 3x, 4x ploidy levels, with aneuploid representatives at all three levels. The species *P. japonicus* displays 4x and aneuploid 8x ploidy, while *P. mauritianus* is predominantly 4x^26^. Significant intraspecific variation was documented in *P. australis* through morphological traits and molecular markers, leading to the proposed recognition of three subspecies: *P. australis ssp. australis*, *P. australis ssp. altissimus*, and *P. australis ssp. americanus*^26,30,31^.

Based on non-coding chloroplast regions *trnT-trnL* and *rbcL-psaI*, researchers have identified 57 *P. australis* haplotypes^32^, with ranges that generally correspond to geographic regions of origin. According to the standard naming system^24^, several phylogenetic studies have found haplotype M as being widely distributed across North America, Europe, and Asia, and subsequent research has confirmed it as an invasive lineage in North America^24^. Haplotype I is found across South America, southern Pacific islands, and Asia while haplotypes U and Q are present in Asia and Australia. Haplotype O is primarily distributed in northern China, and haplotype P is found mainly in Korea and central to southern China. Several unique haplotypes have emerged endemically in Mexico and North America. In southwestern China, three main haplotypes-I, Q, U- can be observed^24,25,33,34^ (Supplementary Table S1). The non-continuous distribution of chloroplast haplotypes within geographic regions complicates the determination of their original spread, particularly when considering the observed variation in intraspecific ploidy levels. Studies based on amplified fragment length polymorphisms (AFLP) and microsatellites have further supported the geographic lineage classifications. However, the AFLP analysis produced only 107 variable sites among the species, which may be insufficient for analyzing allopolyploid species with up to 144 chromosomes^26^.

In United States, the range of exotic *P. australis* has expanded from a restricted area to nearly the entire country within just 50 years, pushing the native lineage to the far north; thus, it is considered as an invasive plant^24^. One hypothesis for the development of invasiveness in *P. australis* is related to polyploidizations^22,35^. Polyploids, with their increased genomic plasticity, may colonize a broader range of habitats^4^. Consequently, successful allopolyploids may become invasive and drive their diploid ancestors to extinction through recurrent hybridizations.

In this study, we aim to test two hypotheses: (1) How does polyploidization influence the genetic diversification process in grass species? and (2) Does hybridization act as a genetic catalyst for polyploidization and contribute to the displacement of native North American lineages in *P. australis*? To address these questions, we first predicted the ploidy level of each individual based on the alternative allele frequencies of reads mapped to the reference genome. Next, we examined the intraspecific genetic divergence, population genetic structure, historical demographics, and hybridization of *P. australis* using RAD-seq approaches. These analyses incorporated data from both the nuclear genome and chloroplast from 88 individuals sampled across the geographic range of the species, with a specific focus on invasive populations and their source populations from Europe.

## Results

### Intraspecific genetic divergence in *P. australis* is associated with polyploidization

**Fig. 1:**
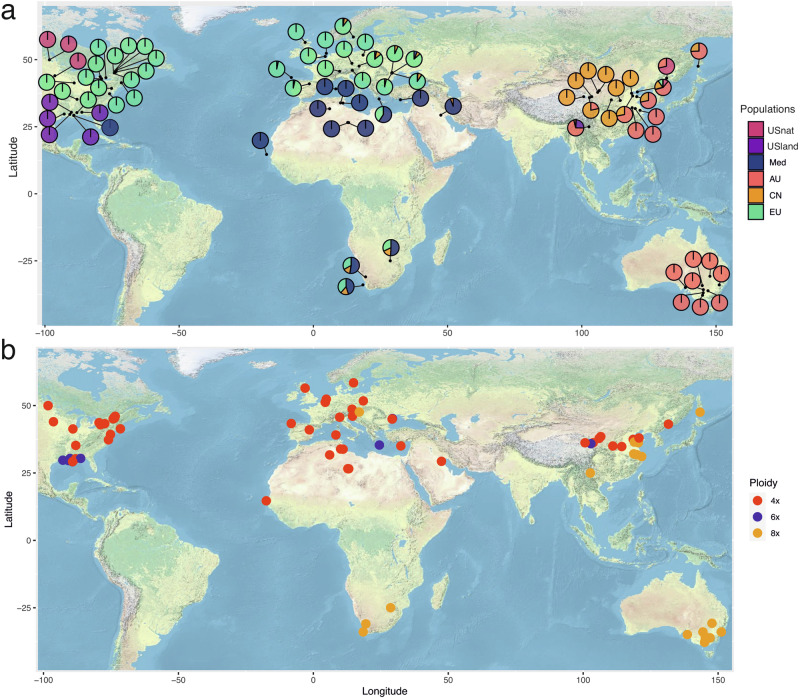
Sampling locations of *Phragmites australis* individuals in the study. **a** Lineage classification and genetic admixture proportions for each sample are presented using pie charts, displayed geographically. Distinct colors indicate genetic lineages. **b** The distribution of each sample by distinct ploidy levels is shown. Ploidy levels were primarily estimated using flow cytometry, and for samples lacking flow cytometry data, predicted values were used. Different colors represent distinct ploidy level.

We carried out RAD-seq on a collection of 88 *P. australis* individuals from the Eurasian, North American, Oceanian and African continents (Fig. 1a, b; Table 1). A subset of these samples was analyzed using flow cytometry to obtain genome size estimates (Table 1). Some of these samples were previously characterized in earlier publications^36^.

**Table 1:** Sampling information and genetic background of the individuals used in RAD-tag sequencing

Sample name	Species	Code	Region	Coordinate	Predicted ploidy	Genome size based on flow cytometry(pg)	Population Grouping	F coefficient
A10	*Phragmites australis*	643	Romania	45°00'00.0“N 29°13'00.0“E	4x	2.01	EU	0.4626290
A2	*Phragmites australis*	602	Holland	52°23'00.0“N 4°49'00.0“E	4x	1.98	EU	0.4874794
A3	*Phragmites australis*	615	Sweden	58°27'00.0“N 14°54'00.0“E	4x	1.89	EU	0.4989708
A5	*Phragmites australis*	663	Hungry	47°36'00.0“N 17°02'00.0“E	8x	3.95	EU	0.7521384
A7	*Phragmites australis*	624	Romania	45°10'00.0“N 29°20'00.0“E	8x	3.91	EU	0.7375464
A8	*Phragmites australis*	625	Romania	45°10'00.0“N 29°20'00.0“E	6x		EU	0.6590505
A9	*Phragmites australis*	WHS4	US	29°12'28“N 89°12'33“W	4x		Med	0.6591026
B2	*Phragmites australis*	651	Romania	45°00'00.0“N 29°13'00.0“E	6x		EU	0.6851450
C10	*Phragmites australis*	131	Canada	45°05'00.0“N 74°11'00.0“W	4x	2.05	EU	0.5537149
C11	*Phragmites australis*	151	Canada	45°30'00.0“N 73°35'00.0“W	4x	2.07	EU	0.6018592
C12	*Phragmites australis*	152	Canada	45°34'00.0“N 73°51'00.0“W	4x	2.16	EU	0.7253990
C13	*Phragmites australis*	153	Canada	45°08'00.0“N 74°00'00.0“W	4x	2.10	EU	0.5612218
C2	*Phragmites australis*	156	Australia	30°45'00.0“S 147°44'00.0“E	8x	4.11	AU	0.6370933
C3	*Phragmites australis*	157	Australia	35°44'15.0“S 145°16'23.0“E	8x	3.91	AU	0.6462146
C6	*Phragmites australis*	167	Australia	36°54'00.0“S 145°14'00.0“E	8x	4.03	AU	0.6586822
C7	*Phragmites australis*	173	Australia	37°49'00.0“S 144°58'00.0“E	8x	4.02	AU	0.6511071
C9	*Phragmites australis*	146	Belgium	51°13'00.0“N 4°25'00.0“E	4x	1.98	EU	0.5090996
D11	*Phragmites australis*	193	US	39°17'48.0“N 75°10'37.0“W	4x	2.03	EU	0.5282259
D12	*Phragmites australis*	154	Canada	46°02'00.0“N 73°26'00.0“W	4x	1.98	EU	0.5797022
D13	*Phragmites australis*	155	Canada	45°14'00.0“N 73°48'00.0“W	4x	2.00	EU	0.5316529
D4	*Phragmites australis*	657	Romania	45°00'00.0“N 29°13'00.0“E	4x	2.02	EU	0.4880390
D5	*Phragmites australis*	8	Italy	39°05'39.0“N 8°21'33.0“E	4x		Med	0.5689966
D6	*Phragmites australis*	987	?	?	4x		EU	0.5329394
D7	*Phragmites australis*	72	Spain	41°00'00.0“N 1°30'00.0“W	4x	1.98	EU	0.5285508
D9	*Phragmites australis*	190	US	42°53'11.0“N 78°52'43.0“W	4x		EU	0.5883302
E12	*Phragmites australis*	204	Canada	49°58'00.0“N 98°18'00.0“W	4x	2.25	USnat	0.9306997
E13	*Phragmites australis*	671	Cyprus	35°02'12.0“N 32°25'35.0“E	4x	1.95	Med	0.5329020
E3	*Phragmites australis*				4x		EU	0.5596739
E9	*Phragmites australis*	102	Dakar	14°40'15.0“N 17°26'17.0“W	4x	1.90	Med	0.6129666
F13	*Phragmites australis*	855	Spain	43°20'36.6“N 8°12'13.2“W	4x		EU	0.5824805
F2	*Phragmites australis*	311	South Africa	24°59'00.0“S 28°38'00.0“E	8x	3.89	admix	0.7088652
F9	*Phragmites australis*	19	Libya	26°35'18.5“N 12°47'15.9“E	4x		Med	0.5298573
H1	*Phragmites australis*	96	Australia	33°58'00.0“S 151°12'00.0“E	8x	4.00	AU	0.6649627
H2	*Phragmites australis*	57	Greece	35°21'53.0“N 24°28'17.0“E	6x		admix	0.6356703
H5	*Phragmites australis*	686	Kuwait	29°19'03.0“N 47°28'47.2“E	4x	1.90	Med	0.5980863
H6	*Phragmites australis*	129	Canada	43°40'00.0“N 79°25'00.0“W	4x	2.09	EU	0.5114880
I12	*Phragmites australis*	97	Tunisia	33°49'00.0“N 11°02'00.0“E	4x	3.92	Med	0.5655735
K12	*Phragmites australis*	183	Russia	43°06'17.0“N 131°35'19.0“E	4x		admix	0.8911014
K13	*Phragmites australis*	215	Russia	47°33'00.0“N 143°19'00.0“E	8x	4.06	admix	0.6993060
K2	*Phragmites australis*	84	Romania	?	6x?	2.97	EU	0.6762899
Y1	*Phragmites australis*	FEAU162	Australia	36°09'00.0“S 147°00'00.0“E	8x	4.04	AU	0.6719352
Y10	*Phragmites australis*	EU207IT	Italy	45°41'00.0“N 9°46'00.0“E	4x	1.94	EU	0.5231192
Y11	*Phragmites australis*	EU60GB	GB	56°27'29.0“N 3°03'03.0“W	4x	1.94	EU	0.5305513
Y12	*Phragmites australis*	LAN126US	US	30°22'43.0“N 90°09'39.0“W	6x	3.09	USland	0.4818650
Y13	*Phragmites australis*	EU620	Czech	48°39'00.0“N 14°22'00.0“E	4x	1.96	EU	0.5409885
Y14	*Phragmites australis*	NAint61	US	41°20'05.0“N 89°06'36.0“W	4x	2.00	EU	0.6399941
Y15	*Phragmites australis*	NAint186	US	37°17'11.0“N 75°55'22.0“W	4x	1.99	EU	0.5887442
Y16	*Phragmites australis*	NAint113	US	41°22'37.0“N 71°30'41.0“W	4x	2.04	EU	0.5315376
Y17	*Phragmites australis*	NAnat211	US	44°00'02.0“N 96°19'02.0“W	4x	2.03	USnat	0.9306310
Y18	*Phragmites australis*	MED174	Tunisia	33°53'00.0“N 10°07'00.0“E	4x	1.92	Med	0.5848370
Y19	*Phragmites australis*	EU172	Slovenia	46°03'19.0“N 14°30'52.0“E	4x	1.96	EU	0.7527237
Y20	*Phragmites australis*	EU67	Belgium	51°13'00.0“N 4°25'00.0“E	4x	1.96	EU	0.4969260
Y21	*Phragmites australis*	MED15	Lybia	26°33'32.0“N 13°07'06.0“E	4x		Med	0.7338246
Y22	*Phragmites australis*	LAND224	US	35°13'22.0“N 88°02'39.0“W	4x	1.97	EU	0.5330414
Y23	*Phragmites australis*	EU78	Poland	51°44'00.0“N 18°31'00.0“E	4x	1.92	EU	0.5324003
Y24	*Phragmites australis*	LAND101	US	30°15'19.0“N 88°06'35.0“W	4x		EU	0.5332007
Y25	*Phragmites australis*	FEAU150	Australia	34°28'00.0“S 146°01'00.0“E	8x	4.00	AU	0.6457727
Y26	*Phragmites australis*	Delta215	US	29°15'34“N 89°14'32“W	4x	2.07	Med	0.6786632
Y27	*Phragmites australis*	NAnat130	US	49°58'00.0“N 98°18'00.0“W	4x	2.25	USnat	0.9150782
Y28	*Phragmites australis*	MED68	Algeria	31°42'07.0“N 6°03'16.0“E	4x	1.97	Med	0.5485428
Y3	*Phragmites australis*	ZA188	South Africa	30°58'00.0“S 19°27'00.0“E	8x	3.95	admix	0.7125974
Y30	*Phragmites australis*	LAND109	US	30°23'45.0“N 86°13'44.0“W	6x	3.12	USland	0.5107852
Y31	*Phragmites australis*	Delta208	US	29°15'17“N 89°14'27“W	4x	1.92	EU	0.6035490
Y32	*Phragmites mauritianus*				4x		CN	0.5363337
Y33	*Phragmites australis*	Land206	US	29°10'16.4“N 89°16'14.4“W	6x		USland	0.5013505
Y34	*Phragmites australis*	DELTA210	US	29°15'14“N 89°14'29“W	4x	2.02	Med	0.6940500
Y35	*Phragmites australis*	Delta144	US	29°44'30.0“N 92°49'18.0“W	6x	3.09	USland	0.4918896
Y36	*Phragmites australis*	ZA105	South Africa	33°56'45.0“S 18°27'46.0“E	8x	3.93	admix	0.7040981
Y37	*Phragmites australis*	LAD118	US	29°35'44.0“N 90°43'10.0“W	6x		USland	0.5104751
Y38	*Phragmites australis*	YRD9	China	37°27'35.6“N 118°32'18.1“E	4x		CN	0.5411762
Y39	*Phragmites australis*	CHANGZHOI	China	31°45'57.9“N 119°55'06.1“E	8x		AU	0.7010347
Y4	*Phragmites australis*	NAint191	US	43°16'35.0“N 77°16'40.0“W	4x	2.02	EU	0.5241846
Y40	*Phragmites australis*	YUNCHENG	China	35°00'38.0“N 110°59'50.1“E	4x		CN	0.5249877
Y41	*Phragmites australis*	GANSU	China	36°12'27.8“N 103°42'08.5“E	4x		CN	0.5479036
Y43	*Phragmites australis*		China	36°12'58.6“N 120°27'45.6“E	8x		admix	0.7306636
Y44	*Phragmites australis*	QH05	China	35°50'44.2“N 102°51'15.5“E	6x		admix	0.6423147
Y45	*Phragmites australis*	QH0B	China	36°12'10.8“N 100°41'20.8“E	4x		CN	0.5160759
Y46	*Phragmites australis*	NX15	China	38°29'27.2“N 106°31'21.7“E	4x		CN	0.5224431
Y47	*Phragmites australis*	NX21	China	37°49'37.9“N 105°55'19.2“E	4x		CN	0.5202388
Y48	*Phragmites australis*		China	24°58'33.2“N 102°40'18.0“E	8x		admix	0.6391666
Y49	*Phragmites australis*		China	36°28'12.2“N 118°56'09.7“E	8x		admix	0.7164914
Y51	*Phragmites australis*		China	32°05'42.5“N 118°44'40.6“E	8x		AU	0.7094003
Y53	*Phragmites australis*		China	31°07'27.9“N 121°41'55.4“E	8x		AU	0.7097670
Y55	*Phragmites australis*	1234	China	37°58'41.9“N 120°41'39.1“E	4x		admix	0.6842193
Y56	*Arundo donax*		China	36°12'58.6“N 120°27'45.6“E	?		OG	0.8527072
Y57	*Phragmites australis*		China	114°17'46.9“E 34°48'04.5“N	4x		CN	0.5704187
Y6	*Phragmites australis*	FEAU136	Australia	34°56'00.0“S 138°36'00.0“E	8x	4.05	AU	0.6671984
Y7	*Phragmites australis*	Land212	US	29°12'25“N 89°12'31“W	6x	3.17	USland	0.5155284
Y9	*Phragmites australis*	213			8x		AU	0.6446644

The code denotes the unique ID shared among PhragNet member groups, which can be used to trace the individuals in other studies. Ploidy was measured using flow cytometry, and predicted ploidy was inferred from the method described in this study. Genetic grouping and F coefficient were inferred with RAD-seq data aligned to the reference genome representing the invasive lineage from North America.

Reference: Meyerson et al. Do ploidy level and nuclear genome size and latitude of origin modify the expression of *Phragmites australis* traits and interactions with herbivores? 2016. Biol Invasions.

### Population genetics analysis reveals links between polyploidization and lineage divergence

**Fig. 2:**
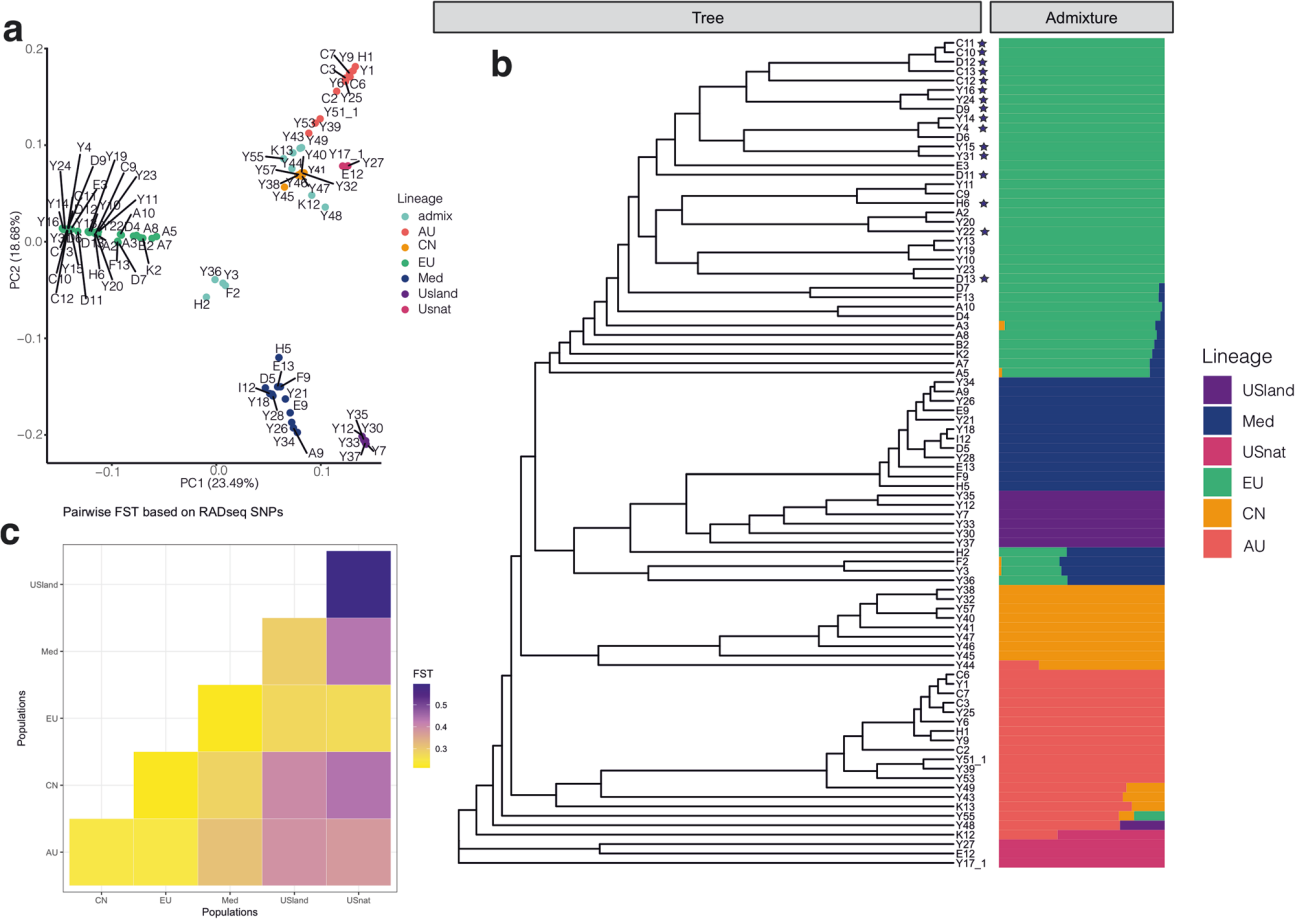
Nuclear phylogeny and population structure of *Phragmites australis* lineages. **a** Population structure of *P. australis* samples using principal component analysis based on variants called against the reference genome of the invasive EU lineage. **b** Left: Maximum likelihood phylogenetic tree estimated from 643,615 variants aligned to the reference genome, with 50% of missing data allowed; Right: fast STRUCTURE population assignments based on variants. The color legends show the six major lineages, and the stars indicate the invasive individuals. **c** Pairwise F*st* values showing population divergence between major lineages of *P. australis*. Darker colors indicate higher level of population differentiation.

A reference-guided alignment against the assembly of the North American invasive lineage^37^ resulted in 1,524,048 loci and 34,997,178 SNP positions. The mean number of sites per locus was 545.41, and the mean coverage of each sample was 26.8x (ranging from a minimum of 15.1x to a maximum of 37.9x). Of these SNPs, 7362 mapped to chloroplast genome. After selecting one random SNP from each RAD-seq locus, we obtained a total of 643,615 variant sites. Following filtering for loci with no more than 50% missing values and a minor allele frequency greater than 5%, 90,808 SNPs remained. Maximum likelihood phylogenetic trees revealed six distinct genetic clusters with high bootstrap confidence values (Supplementary Fig. S1), namely the North American native lineage (USnat), Australian lineage (AU), Chinese lineage (CN), European lineage (EU), South African lineage, Mediterranean lineage (Med), and US land type (USland) (Fig. 2b; Supplementary Fig. S1). Population structure analyses using fastSTRUCTURE supported six as the best number of ancestral populations based on the highest marginal likelihood, aligning with the splits in the phylogenetic tree, with the exception of the South African lineage (Fig. 2b). Admixed individuals were mainly detected between the AU and CN lineages, the EU and CN lineages, and among the Med, EU and CN lineages (Table 1; Supplementary Fig. S2).

**Fig. 3:**
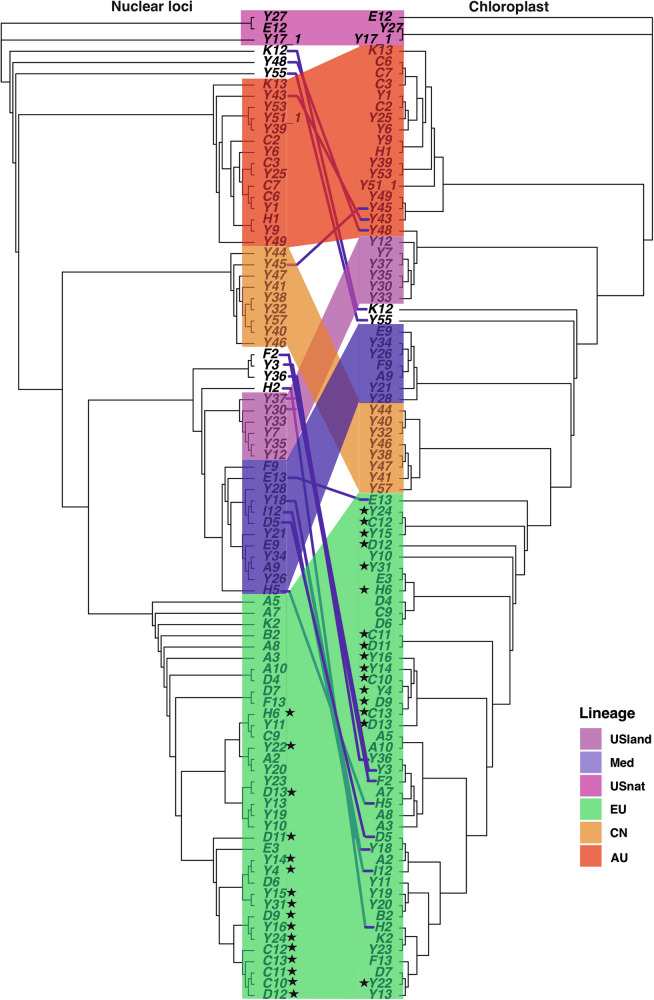
Comparison between nuclear and chloroplast phylogenies. The left figure showed rooted phylogenetic tree constructed from reads mapped to nuclear reference genome. The right figure showed rooted phylogenetic tree constructed from reads mapped to the chloroplast genome. Both trees were rooted using the North American native lineage. Branch colors indicate the genetic groups corresponding to nuclear trees. The stars indicate the invasive individuals. Lines between the trees connect the same individuals among the two trees.

The six genetic groups were further supported by the phylogeny estimated from chloroplast SNPs, with varying bootstrap values. Approximately 13 individuals exhibited discordant placements between chloroplast and nuclear phylogenetic trees, likely due to chloroplast capture events, suggesting extensive intraspecific gene flow. For example, five individuals from the Med lineage carry chloroplasts of the EU lineage, while another seven genetically admixed individuals carry chloroplasts of a group intermediate between the main genetic groups (Fig. 3).

The genetic groups were showing clear geographic distribution: the USnat lineage is distributed at the border between the United States and Canada, while the USland type is located around the Gulf of Mexico. The Med lineage is present in Mediterranean regions, North Africa, and the Gulf of Mexico. The CN lineage is mainly located along the Yellow River in China, and the AU lineage is present in Australia, Southern China, the Pacific peninsula and islands, and the Korean peninsula. The EU lineage is widespread across Europe and also represents the invasive lineage in the United States (Fig. 1). Finally, the CN clade is largely confined to the Yellow River watershed, suggesting that this lineage may have originated in western China and subsequently spread along the river to eastern China.

Although we were unable to obtain the sequences of *trn*T-*trn*L and *rbc*L–*psa*I regions to directly compare with previous studies based on chloroplast haplotypes^24,34,38^, we inferred the corresponding haplotypes based on geographic locations (Supplementary Table S1). For example, the chloroplast haplotype P, which is distributed in Eastern China, may correspond to the AU lineage due to their overlapping geographic occurrences^28,39^. Representatives of the invasive lineage from North America grouped together with the EU lineage in the nuclear phylogenetic tree, consistent with the documented history of artificial transfer from EU to North America within a short time frame^24,38^. In the chloroplast phylogenetic tree, these invasive individuals were paraphyletic and placed in several subtrees of the EU lineage, suggesting multiple introductions to North America (Fig. 3).

#### Multiple ploidy levels were observed in *P. australis*

**Fig. 4:**
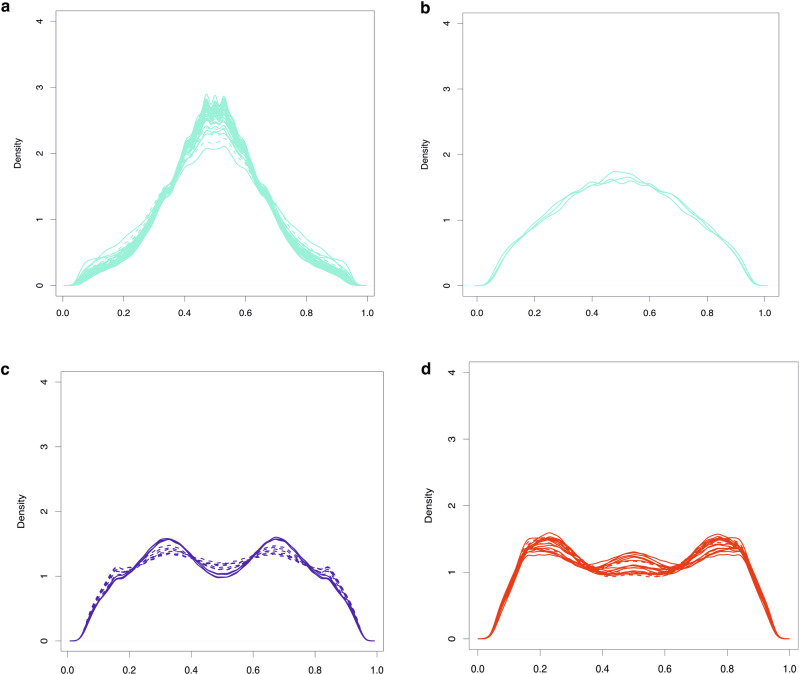
The alternative allele frequency histograms from aligning the RAD-seq reads to the reference genome. Solid line shows frequency distribution of alternative alleles in individuals where the result was confirmed with flow cytometry. Dashed line shows frequency distribution of alternative alleles in individuals with missing or differing flow cytometry measurement. Turquoise, red, and blue color represented tetraploid, hexaploidy, and octoploid respectively. Pattern of alternative allele frequency in population tetraploids (**a**), USnat (**b**), hexaploids (**c**), and octoploids (**d**).

For RAD-seq data, a histogram of the proportion of reads supporting the alternative allele revealed modes at around 0.5 for flow cytometry-confirmed tetraploids, at 0.35 and 0.65 for hexaploids, and between 0.35 and 0.65 for octoploids (Fig. 4). These proportions indicate that the RAD-seq loci were unique to the two subgenomes of the allotetraploid common reed. Out of the 88 individuals, the ploidy levels of 64 were quantified using flow cytometry. Only three samples (Y24, E3, Y37) showed discrepancies between the flow cytometry and our read-based prediction method, which may be attributed to mislabeling of samples or potential aneuploidy. Therefore, the prediction accuracy is at least 95.3% (Table 1), demonstrating that the prediction of ploidy levels from RAD-seq data can be done accurately.

We predicted the ploidy levels for the remaining 24 individuals using the in silico method. Most individuals from North America, Northern China, Mediterranean regions, and Europe (representing the USnat, CN, Med, EU lineages; see Fig. 4) were predicted to be allotetraploid, with a unimodal distribution peaking at 0.5 (Table 1; Fig. 4a, b). All representatives from the Gulf Coast (USland lineage) and several admixed individuals, were predicted to be hexaploids (Fig. 4c). In contrast, all Australian (AU lineage) individuals and a few admixed individuals from South Africa and the Mediterranean region were predicted to be octoploids (Fig. 4d).

In summary, each lineage detected in the population structure analyses was characterized by its own ploidy level, and the phylogenetic tree suggests different origins of polyploidization. Specifically, the AU lineage is auto-allo-octoploid (genome doubling of the allotetraploid), the US land lineage is allohexaploid resulting from interspecific hybridization, and the South African population is auto-allo-octoploid (genome doubling of the allotetraploid, Supplementary Fig. S3). The other lineages such as CN, Med, and most EU individuals are allotetraploids.

Out of 16 admixed individuals identified by fastSTRUCTURE, 14 showed increased ploidy levels and two showed decreased ploidy levels compared to parental populations. Five individuals, including samples from South Africa, Romania, and Hungary, were admixed offspring of tetraploid lineages CN, EU, and Med (Table 2), but were found to be octoploid. Additionally, three individuals with similar backgrounds were predicted to be hexaploids; we refer to these individuals as Neoploid (Balkans), including samples Y3, Y36, A5, A7, A8, B2, F2, H2, and K2 (Table 2). The octoploids from the Danube delta were previously classified into the EU lineage^40^. The formation of new ploidy levels through inter-population hybridization of lower ploidy levels has also been reported in *Arabidopsis*, suggesting that this mechanism is not uncommon in the plant kingdom^41^.

**Table 2:** Predicted ploidy levels for admixed individuals

Individual ID	Admixture lineages	Ratio of admixture	Ploidy of original lineages	Expected ploidy in F1 of parental lineages^a^	Expected ploidy^a^	Predicted ploidy^b^	Flow cytometry estimate	Location
Y44	AU/CN	0.24/0.76	4x/8x	6.0	5.0	6x	?	China
K13	AU/CN	0.80/0.20	8x/4x	6.0	7.2	8x	8-10x	Russia
Y43	AU/CN	0.75/0.25	8x/4x	6.0	7.0	8x	?	China
Y49	AU/CN	0.77/0.23	8x/4x	6.0	7.1	8x	?	China
Y48	AU/USland	0.73/0.27	8x/6x	7.0	7.5	8x?	?	China
Y55	AU/CN/EU	0.72/0.09/0.18	8x/4x/4x	6.0	6.8	4x	?	China
Y3	Med/EU/CN	0.62/0.36/0.01	4x/4x/4x	4.0	4.0	8x	8x	South Africa
F2	Med/EU/CN	0.63/0.35/0.01	4x/4x/4x	4.0	4.0	8x	8x	South Africa
H2	Med/EU	0.59/0.41	4x/4x	4.0	4.0	6x	6x	Greece
Y36	Med/EU	0.59/0.41	4x/4x	4.0	4.0	8x	8x	South Africa
K12	USnat/AU	0.65/0.35	4x/8x	6.0	5.4	4x	4x	Russia
A7	EU/Med	0.91/0.09	4x/4x	4.0	4.0	8x	8x	Romania
K2	EU/Med	0.92/0.08	4X/4X	4.0	4.0	6x	6x	Romania
A8	EU/Med	0.95/0.05	4X/4X	4.0	4.0	6x	6x	Romania
B2	EU/Med	0.94/0.06	4X/4X	4.0	4.0	6x	6x	Romania
A5	EU/Med/CN	0.89/0.09/0.02	4X/4X/4X	4.0	4.0	8x	8x	Hungray

^a^Expected ploidy is calculated from Weighted arithmetic mean.

^b^predicted ploidy is inferred from the alternative allele frequency.

#### Ploidy levels and geography explain the population structure

We next carried out principal component analysis (PCA) of the nuclear RAD-seq dataset to determine whether different ploidy levels are distinguishable by SNPs. The first three principal components explained 52.1% of the variance in the data and showed segregation patterns according to geography and ploidy level. Hexaploids were predominantly positioned between the tetraploid and octoploid population groups (Fig. 2a). The first PCA axis separated EU tetraploids, USland hexaploids, and South African octoploids from other groups, while the second axis further distinguished Med tetraploids from AU octoploids and CN tetraploids (Fig. 2a). Although AU and CN lineages differed in their ploidy levels, they were not clearly separable in the PCA plot.

**Fig. 5:**
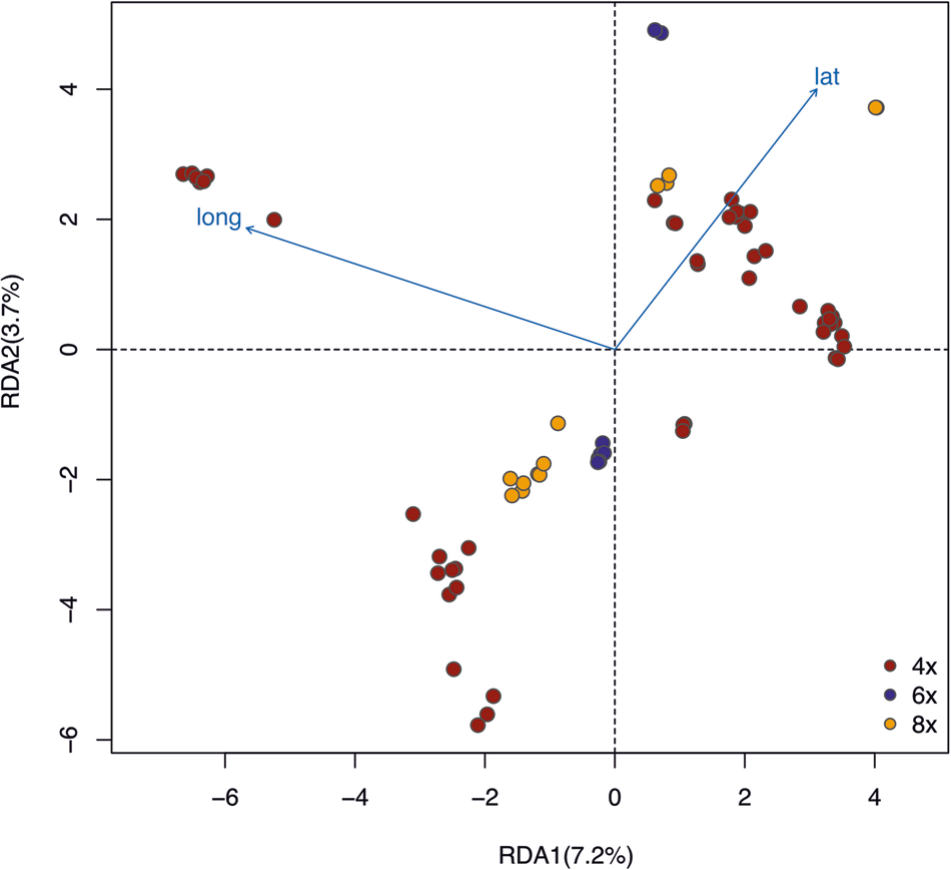
RDA analysis under constraint model using coordinates as explanatory variables and ploidy level as covariates. RDA1 explains 7.2% of the data, and RDA2 explains 3.7% of the data. The arrows lat and long indicated the latitude and longitude of sampling sites of each individual. Ploidy level explains 15.09% of the variance and latitude and longitude explains 7.29% of the variance.

To examine the sources of genetic variation more precisely, we carried out a redundancy analysis (RDA; Fig. 5)^42,43^. Altogether 44.97% of the total genetic variation was explained by the genetic groups, whereas ploidy levels accounted for 15.09% of the variation. Geographic coordinates contributed a small but significant proportion of 2.5% of variance (latitude, *p* = 0.01; longitude, *p* = 0.01, respectively) after modeling the effect of population group as a covariate. Ploidy levels were confounded within the population groups, and thus did not explain any additional variance beyond that explained by the groups.

According to the leading-edge hypothesis, source populations retain more genetic diversity than populations in newly colonized areas during species spread^44,45^. To test whether the spread routes of *P. australis* populations align with this hypothesis, we compared geographically isolated individuals from the same genetic group. For the pure EU lineage, nucleotide diversity in invasive North American populations originating from Europe (0.15475, *n* = 16) was much lower than in source populations within Europe (0.24777, *n* = 36), in support of the known founding event. In the AU lineage, nucleotide diversity in the Southeastern China population (0.27332, *n* = 3) was slightly lower than in Australian populations (0.27975, *n* = 8). The similar diversity levels suggest that the AU lineage may be widespread throughout the Asian continent. However, the number of private alleles was much higher in Southern China populations (364,298) compared to Australian populations (201,898), suggesting that the populations in China may have a higher population growth rate than the populations in low-latitude regions and Australia. Tetraploid lineages of *P. australis* exhibited a gradient in genetic diversity, decreasing from EU (0.17756, *n* = 36) to Med (0.14095, *n* = 12), CN (0.14057, *n* = 8) and USnat (0.038091, *n* = 3). This pattern suggests that the core lineage of *P. australis* may have originated in temperate grasslands of the former Laurasia (see ref. ^46^ for a reconstruction of a late Miocene vegetation map), and subsequently diverged into Asian, European, and Mediterranean populations.

#### Extensive gene flow in *Phragmites australis*

Based on our phylogenetic analyses, the USnat lineage diverged early from the main Laurasian population. Consequently, genetic divergence between USnat and other lineages was high (F_*st*_ > 0.27, Fig. 2c), especially with USland, see refs. ^27,47^. The USland lineage showed high divergence from CN (F_*st*_ = 0.40) and showed moderate divergence with EU and Med (0.28 < F_*st*_ < 0.29). The lowest F_*st*_ values were observed between EU and Med, as well as between EU and CN lineages (0.213), suggesting more recent divergence and gene flow (Fig. 2c). We therefore analyzed gene flow using formal tests of introgression.

**Fig. 6:**
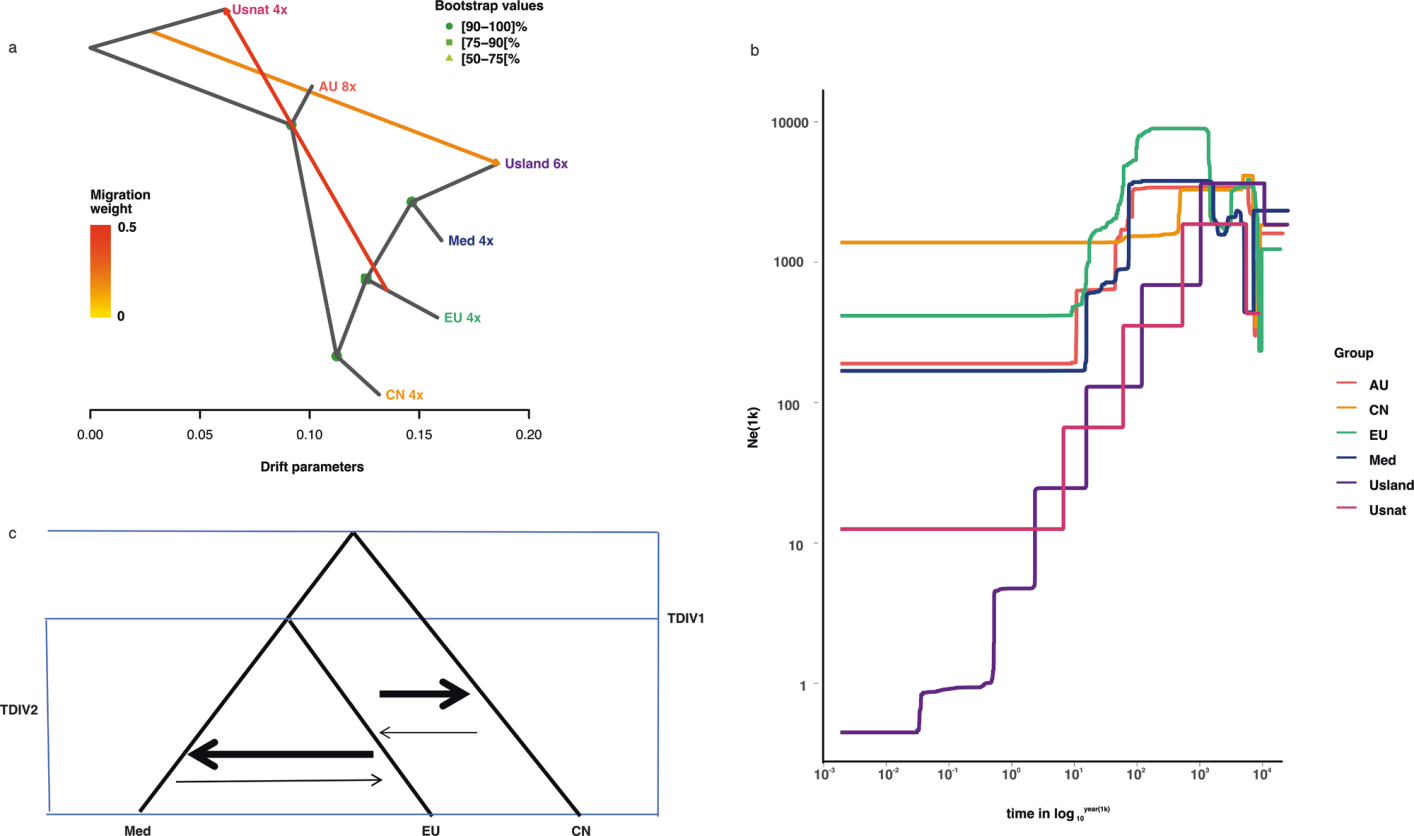
Gene flow and historical demographic scenarios of each lineage. **a** Two migration events inferred with Treemix indicate that one ancestor for USland lineage may be an unsampled species genetically close to USnat. Gene flow occurred from an early population of EU to USnat. **b** Historical effective population sizes of six *P. australis* lineages estimated with Stairway plot (AU, CN, EU, Med, USland, and USnat). Admixed individuals were excluded from the population analyses. **c** The best demographic model estimated from pairwise derived allele site frequency for the three core tetraploid lineages. This is inferred with fastsimcoal2 with mutation rate of 2.17e^–09^ substitutions per site per year and generation time of 4 years.

To trace the origin of higher ploidy levels, we conducted f3 tests to investigate whether octoploids and hexaploids were sourced from hybridization among different populations within the same species. In an f3 test, a negative value for *f*3(Pop1; Pop2, Pop3) indicates that Pop1 is admixed between populations related to Pop2 and Pop3. We tested all combinations of the six populations and found that none of the f3 tests yielded negative values, providing no evidence of hybridization (Supplementary Table S2; Supplementary Fig. S4). Population migrations estimated with Treemix were consistent with the topology of the maximum likelihood phylogenetic tree. A model incorporating two migration events received the highest support from the data (Supplementary Fig. S5). This model suggests one migration event from an ancestral lineage genetically related to the USnat lineage into the USland lineage, alongside a more pronounced migration from the ancestral population of the EU lineage into the USnat lineage (Fig. 6a). This suggests that one of the ancestors of the hexaploid USland lineage may be a ghost lineage not sampled in our study. In fact, the USland lineage, habituated in the Gulf Coast of North America, has been identified as a hybrid between Mediterranean *P. australis* and *P. mauritianus*^27^. Similar to Mediterranean *P. australis*, the USland lineage shows significantly higher photosynthetic efficacy than the EU lineage, which is probably an adaptive mechanism to its origins in tropical Africa and the Mediterranean area^48^. Additionally, previous studies have identified a 200 bp band in the *waxy* gene of the USland lineage from *P. australis*, and a 100 bp DNA fragment specific to *P. mauritianus*^27^, confirming its hybrid status. Hybridization between *P. australis* and *P. mauritianus* has also been observed in Southern Africa in recent decades^49^. In the nuclear phylogeny, USland is grouped with the Med lineage (Fig. 2b), supporting the hypothesis that the Med lineage is probably the second parental lineage of USland. Conversely, the USland lineage groups together with the AU lineage in the chloroplast phylogenetic tree, suggesting that the chloroplasts of the USland lineage may have been introgressed from the other parental species during interspecies hybridization events that took place prior to the emergence of the AU lineage (Supplementary Fig. S6).

The fast STRUCTURE results revealed that more than 18% of all individuals were admixed, a finding consistent with the incongruence observed between nuclear and organellar phylogenies. The f4 tests, using USnat as the outgroup, supported gene flow between most lineages, except for the USland and CN lineages, as well as between USland and EU lineages (Fig. 2b; Table 2, Supplementary Table S3). We next carried out population divergence time modeling using Approximate Bayesian Computation in Fastsimcoal2, fitting joint site frequency spectra (SFS) two-population split models. Given that the estimation of site frequency spectrum in polyploids can complicate the inference of demographic history, we restricted our simulations to the allotetraploids. We simulated four divergence-with-migration scenarios involving the three core tetraploid lineages (CN, EU, and Med), and one scenario including USnat along with these core lineages (Supplementary Fig. S7). We examined five scenarios to model population divergence and gene flow: Scenario 1 assumed constant gene flow between the geographically overlapping EU and Med lineages; Scenario 2 included constant gene flow between EU and Med lineages, as well as ancient gene flow between CN and the common ancestor of EU and Med lineages; Scenario 3 involved constant gene flow among CN, EU, and Med; Scenario 4 assumed no migration; and Scenario 5 assumed no migration with an exponential population decline of the USnat lineage. The runs with the highest likelihood for each scenario were selected. Scenario 3 yielded the lowest AIC values and smallest likelihood difference between the observed data and simulated parameters (Supplementary Table S4). This scenario estimated that it takes 166,063 generations for Med and EU to coalesce into their most recent common ancestor (MRCA), and 168,092 generations for this MRCA to further coalesce with the CN lineage. These estimates correspond to divergence times of 0.332–0.664 Mya between EU and Med, and 0.396–0.672 Mya between CN and MRCA of EU and Med, based on a generation time of 2–4 years. These estimates are considerably younger than those obtained with single copy BUSCOs (Benchmarking Universal Single-Copy Orthologs), which estimated the divergence at 2.2–3.35 Mya^50^. The discrepancy is likely due to the limited number of samples and segregating sites in the RAD-seq data. The migration matrix showed greater migration intensity from CN to EU (2.379 × 10^−6^) compared to EU to CN (1.132 × 10^−6^), and from Med to EU (3.905 × 10^−6^) compared to EU to Med (8.698 × 10^−7^) when viewed backwards in time. Conversely, when viewed forwards in time, the pattern is reversed, showing greater levels of introgression from EU to CN, and from EU to Med than in the reverse directions (Fig. 6c).

### Hybridization and polyploid diversity

For RAD-seq data, nucleotide diversity (π) was estimated to be highest in the EU lineage (0.17756, *n* = 36), and lowest in USnat lineage (0.038091, *n* = 3) (Table 3). The other lineages showed intermediate levels of polymorphism, with π ranging between 0.11673 and 0.15987 (Table 3). The inbreeding coefficient (F) was positive for all accessions, being highest among the USnat lineage and lowest in the USland lineage, suggesting a higher level of inbreeding in USnat and relatively more outbreeding in USland (Table 3). Tajima’s D value was positive for all populations, being moderately high in AU (0.26 ± 0.86), EU (0.53 ± 0.82), CN (0.06 ± 0.91), Med (0.32 ± 0.86) and USnat (0.54 ± 0.92) lineages, and exceptionally high in USland lineage (1.04 ± 0.82) (Table 3). Low nucleotide diversity combined with high Tajima’s D values are in general hallmarks of a population bottleneck, and we therefore continued by assessing the population history of the species. Using Stairway plot based on the site frequency spectrum from RAD-seq data and assuming a generation time of four years, we detected an early population increase between 1 and 5 Mya for USnat, AU, CN, and EU lineages (Fig. 6b) This was followed by a recent decline in effective population sizes across nearly all the lineages. The decline in the AU, Med, and EU populations started from 50 Kya. The USland population experienced a dramatic decline throughout its history, starting at approximately 800 Kya. The USnat population began a continuous decline around 700 Kya.

**Table 3:** Summary of genetic variation in each of the *P. australis* lineages

Lineage	No. Samples	Variant sites	Nucleotide diversity (π)	Inbreeding coefficient (Fis)(mean ± SD)	Tajima’s D (mean ± SD)
AU	12	484322	0.15987	0.67 ± 0.03	0.26 ± 0.86
CN	8	441226	0.14057	0.53 ± 0.02	0.06 ± 0.91
EU	36	529629	0.17756	0.55 ± 0.06	0.53 ± 0.82
Med	12	438879	0.14095	0.61 ± 0.07	0.32 ± 0.86
USland	6	378885	0.11673	0.50 ± 0.01	1.04 ± 0.82
USnat	3	275581	0.038091	0.92 ± 0.02	0.54 ± 0.92

#### Validation of population genetic statistics for polyploids

Polyploids present more uncertainty for genomic variants. Therefore, genotype calling, and SNP filtering processes designed for diploids may not be directly applicable to polyploids. Since most pipelines are developed for diploids and most of our samples are diploidized allotetraploids, we initially conducted our analyses using a diploid pipeline and then verified the results for the individuals of higher ploidy levels. We compared the inbreeding coefficient of all individuals calculated using diploid analysis software (Plink) with the values calculated using polyRAD, a software considers ploidy levels in heterozygosity estimation. Similar values were obtained between the two analyses, suggesting that the number of loci which could potentially bias the heterozygosity did not account for a large proportion of the data. The heterozygosity and population structure inferred using genotypes called from polyRAD were consistent with our conclusions, indicating that our results based on filtered biallelic loci are robust. However, demographic analyses involving the AU lineage and other polyploid lineages resulting from admixture should still be interpreted with caution.

## Discussion

In this study, several polyploids were identified in *P. australis*, and the geographic distribution of ploidy levels reflects distinct genetic lineages, suggesting that independent polyploidization events may have driven species diversification. These polyploidization events are often associated with either interspecific or intraspecific hybridization, leading to the formation of both autopolyploids and allopolyploids. This indicates that genetic conflicts among the genomes of *Phragmites* species may be effectively resolved through genome doubling. This is reflected by the hexaploids and octoploids discovered in South Africa, Romania, and its surrounding areas, which formed due to intraspecific hybridization, as well as the USland lineage, which involved interspecific hybridization. A similar pattern was observed in *Miscanthus sacchariflorus*, a grass species with three diploid lineages in China and Russia, and three tetraploid lineages in South Korea and Japan^51^. One of the tetraploid lineages, distributed along the Chinese coast and in South Korea, was genetically admixed with the diploid lineages in China, while other tetraploid lineages exhibited introgression from *M. sinensis*. This suggests that hybridization and whole genome duplication may be substantial drivers in shaping the high genetic diversity and speciation process in grass species. The invasive populations of common reed have split from their ancestral populations less than 200 years ago and are thus genetically still part of the EU lineage. Similar to the other individuals in EU lineage, they are still allotetraploids, and therefore polyploidization is not a causal factor for invasiveness.

The divergence of the three allotetraploid lineages in *P. australis* is relatively recent, indicating rapid genomic changes associated with lineage radiation. Extensive gene flow was observed among multiple lineages through D statistics, Treemix, fastsimcoal, and population structure analyses, highlighting the existence of multiple secondary contact zones. These contact zones provide increased opportunities for intraspecific hybridization. Our data revealed limited admixture between USnat and other lineages, indicating a potential reproductive barrier between them. Consequently, the lack of gene flow, higher level of inbreeding, and subsequent lower fitness likely contributed to the divergence and potential extinction of the North American native lineage.

The octoploid lineages occurring in South Africa and the Balkans are derived from the admixture of the tetraploid Med and EU lineages (Fig. 3; Table 2). The neopolyploids are unique because although the parental lineages are tetraploid, the resulting offspring are either hexaploid or octoploid. Three out of four individuals from South Africa were admixed by Med, EU and CN tetraploid lineages. All three samples, located far from each other, were predicted to be octoploids (Fig. 3), consistent with earlier records^22^. Admixed octoploid individuals were also found in Hungary and Romania; these two populations were clearly distinct in PCA (Fig. 2a), suggesting independent polyploidization events caused by intraspecies genomic conflicts. However, not all admixed individuals experienced chromosome doubling (Table 2). Consistently, intensive investigation has shown the presence of a mix of octoploids, tetraploids, and hexaploids in the Danube Delta, likely originating from recent polyploidization and hybridization^16,40^. Previous studies showed seeds from the same inflorescence could produce offspring with different ploidy levels, suggesting the hexaploidy may result from the hybridization of tetraploid and octoploid parents^52^. A similar phenomenom occurred in *Spartina*, where the dodecaploid *S. anglica* arose from whole genome duplication following the hybridization of two hexaploid species *S. alterniflora* and *S. maritima*. This was followed by recurrent hybridization with one of the parental species *S. alterniflora*, giving rise to nonaploid individuals^53^.

Lineages such as CN, Med, EU, and AU have experienced slight population declines in history, but their populations have remained relatively large. The USland lineage, characterized by red, woody, branched stems, has a limited distribution between inland Texas and Florida^27^. It experienced a significant population decline throughout its history, likely attributable to its low reproductive success as a hybrid lineage^47^. Consequently, the lineage tends to rely more on clonal propagation than sexual reproduction, leading to reduced genetic diversity within the population. Similarly, the USnat lineage has been observed to have a high rate of deleterious mutations and is inferred to rely more heavily on clonal propagation compared to other lineages^50^, which could explain its declining population size.

Autopolyploidization and allopolyploidization are recognized as the primary mechanisms of polyploidization. However, the underlying mechanism of how whole genome duplication happens is still largely unknown^54^. When the parental species are highly divergent, genomic incompatibilities resulting from hybridization may increase the likelihood of errors during meiosis, thereby facilitating the formation of new polyploids. The neopolyploids from South Africa, Romania, Greece, and Hungary exhibit genetic admixture with EU, CN, and Med lineages, suggesting that genome organization and regulatory networks may be disrupted prior to polyploidization. One potential explanation for this is frequent misregulation due to stressful environmental conditions. Extreme environments are known to induce polyploidization, particularly in autopolyploids. For instance, in the Iberian Peninsula, the distribution of diploids and allotetraploids of *Brachypodium distachyon* correlates significantly with environmental dryness; arid conditions may cause issues with cytotype segregation, thereby promoting polyploidization within Poaceae species^55^. In *P. australis*, octoploid occurrences are predominantly found in regions with consistently high temperatures, such as Australia, South Africa, and areas between Central Europe and the Black Sea. The intense, dry, and hot climates in these regions may induce meiotic errors leading to the production of unreduced gametes. This increased plasticity could enhance the species’ ability to adapt to new ecological niches^54,55^. However, this hypothesis is controversial since an experimental study on *Brachypodium* species sampled along an aridity gradient in Israel did not support the notion that arid conditions increase the likelihood of allopolyploidization^56^.

Since these polyploids were observed mainly along the contact zones, a possible genomic component contributing to their formation could be related to the extensive clonal propagation in *P. australis*. The clonal reproduction may lead to relaxed selection and the accumulation of deleterious alleles. Over time, the accumulation of these alleles in allopatric populations may have led into increased genomic divergence, which can cause intraspecific genomic conflicts among progeny from these diverged populations.

## Methods

### DNA extraction, library preparation and RAD sequencing

A total of 88 *P. australis* individuals were obtained from Eurasian, North American, Oceanian and African continents (Fig. 1; Table 1) and planted in Aarhus University and Shandong University. Some of the individuals have already been used in previous phylogeographic studies, allowing us to correlate them with lineages defined in those studies (see more in Table 1). An individual of *Arundo donax* (giant reed) was included as an outgroup. For RAD sequencing, DNA was extracted from fresh leaves using the CTAB method. The leaves were ground into a powder and mixed with 500 μl extraction buffer (containing 100 mM Tris pH 8, 1.4 M NaCl, 20 mM EDTA, 2% CTAB) and 2% β-mercaptoethanol. After incubation at 65 °C for 10 min, the suspension was mixed with 500 μl of chloroform and centrifuged at 10,000 rpm for 5 min. Then, 500 μl of chloroform was added to the supernatant, and the mixture was centrifuged at 10,000 rpm for 10 min. Subsequently, 800 μl of ice-cold ethanol was added, and the tube was left in the freezer overnight. The sample was then centrifuged at 13,000 rpm at 4 °C for 20 min, followed by washing with 500 μl 70% ethanol. The pellet was air dried, centrifuged at 6000 rpm at 4 °C, and then dissolved in 200 μl of H_2_O. Paired-end RAD-seq library preparation and sequencing were performed by Shanghai Honsunbio Limited company, using the Illumina HiSeqX10 platform. Illumine reads of *Oropetium thomaeum* (SRR2083762), *Miscanthus sinensis* (SRR486617), *Arundo donax* (SRR4319201), *Arundo plinii* (SRR4319202) and *Sorghum bicolor* (SRR12628364) were used to construct ancestral alleles of *Phragmites* lineages.

### Variant calling

Since the subgenomes of allotetraploid *P. australis* diverged at around 30.9 Mya, and the tetraploid genome has undergone extensive diploidization^50^, the allotetraploids can be considered as new diploids for subsequent analyses. RAD-tag sequences were processed following the Stacks pipeline with refmap methods, after aligning the reads to a reference genome assembled for the North American invasive lineage using bwa^37,57^. This genome assembly was refined by removing duplicated haplotigs using Purge Haplotigs^58^. We first demultiplexed the sample reads and removed the barcodes using process_radtags function from Stacks^57^. In the refmap pipeline, paired reads were aligned to the purged genome assembly using Bowtie2^59^. To ensure the accuracy of variant calling, only reads uniquely mapped to genomic regions were considered. RAD-seq data were also aligned to a published chloroplast assembly (accession number: KJ825856), to extract genetic information from organelles, and compare the evolutionary histories of nuclear and chloroplast genomes. Since we are analyzing allotetraploid alongside other polyploids, genotype calling was performed using the allotetraploid/diploid pipeline. To account for the effects of polyploidy and validate our population genetic statistics, genotyping for octoploids and hexaploids was specifically conducted using PolyRAD^60,61^. The vcf file produced by Stacks, including allelic read depth information, was used as input for PolyRAD. Population parameters such as heterozysosity and F coefficients were estimated, and genotype calling was performed using Bayesian methods.

### Ploidy level prediction

Although the ploidy level of most individuals was determined using flow cytometry, the knowledge of genome size of all individuals would provide a clearer understanding of the evolutionary pathways of polyploidization. To investigate the relationship between genomic alleles and ploidy levels, we developed a method that predicts ploidy level by analyzing the proportions of alternative alleles based on reference guided aligned reads. After mapping the short reads to the reference genome, similarly but differently from ploidyNGS^19^, we count the number of alleles that are different from the reference genome. We set the total read coverage to range from 20 to 200, considering only alleles with coverage greater than 7 as variants. The number of chromosome copies were inferred by counting the peaks of alternative allele proportions. For example, in diploid individuals with two copies of alleles, the proportion of reads supporting alternative alleles should be ~0.5 at heterozygous sites, resulting a single peak at 0.5 in the genomic distribution of alternative allele proportions. Similarly, in tetraploid individuals, the alternative allele proportions should display peaks at 0.25, 0.5, 0.75, while in hexaploids, peaks would be observed at 0.167, 0.33, 0.5, 0.67, and 0.83 among others. Based on this pattern, we predicted the ploidy levels by plotting the density of alternative allele frequencies and validated the predictions using the flow cytometry data. The analysis involved counting the read depth of alternative alleles in bam files after aligning the filtered reads to the reference genome.

### Phylogeny

For further analyses, a filtered set of high-quality SNPs was obtained by selecting SNPs that are present in more than 50% of the samples using vcftools^62^. Phylogenetic trees were constructed for these filtered SNPs using RAxML^63^ with the GTRGAMMA substitution model and 100 bootstrap replicates. The final tree was annotated and viewed using Figtree (http://tree.bio.ed.ac.uk/software/figtree/). To obtain the chloroplast phylogeny, RADseq reads were aligned to the chloroplast genome assembly (NCBI, accession number: NC_060780). A total of 7362 variants were retained after removing unmapped and supplementary reads. Ambiguous sites in chloroplast alignment may result from organelle introgression into the nuclear genome or organelle capture from different genetic lineages. Consequently, we compared the chloroplast phylogenetic patterns between datasets that included and excluded these ambiguous sites.

### Population structure and reticulate evolution

To evaluate the genetic ancestry of each independent allele and minimize the effect of linkage disequilibrium, we selected the first SNP from each stack locus. Sites with more than 50% missing data were removed, and only biallelic sites with minor allele frequency higher than 0.05 were retained, resulting in a final dataset of 92,769 variants. Population structure was estimated using fastSTRUCTURE with K values ranging from 2 to 15^64^. The optimal K was chosen based on the highest marginal likelihood, and the admixture plot was visualized using the pophelpler^65^ R package. To verify the results, we reprocessed the data by removing sites with more than 50% missing data using vcftools v 0.1.16^62^, and filtering out variants with a minor allele frequency less than 0.05 using Plink 1.9^66^. The processed dataset was then used to perform genetic clustering with fineRADstructure^67^.

PCA was calculated using Plink 1.9^66^ and plotted in R to illustrate the ordination of the genetic data. In addition, we performed RDA using the R package “vegan”^68^ to quantify the proportion of genetic variation explained by covariates, including lineages, geographical location, and ploidy level. To evaluate the significance of the fitted models, 100 permutations were applied.

To understand the genomic structure of each lineage, individuals were grouped into populations based on results from fastSTRUCTURE and phylogeny analysis (Table 1). The F coefficients for all individuals were calculated with the formula 1 - hindhe * ploidy / (ploidy - 1) using polyRAD^60^. Nucleotide diversity (π), private alleles (N_A_) and genetic divergence between populations (F_*st*_) were calculated using the populations program in Stacks^69^. Nucleotide diversity (π) for each individual was assessed using vcftools v 0.1.16 with 1000 bp window size. Based on clustering results, individuals with over 90% of their genotypes assigned to the groups were included in the analyses. To test for admixture, f3 tests were performed using Admixtools 2^70^. With ABBABABA considered significant only if the absolute value of the Z score exceeded 3. To evaluate potential gene flow between lineages, we selected four pure individuals from each lineage (excluding the USnat lineage where one individual was used as an outgroup) and performed ABBABABA statistics among all combinations using AdmixTools v7.0.1^70^. Tajima’s D of each population was measured using vcfkit^71^ using non-overlapping sliding windows of size 10,000 bp to assess population demographics across the genome.

### Population demographics in history

The SFS of derived alleles was utilized in Stairway plot^72^ to infer historical population size changes. Although *P. australis* typically sprouts and matures wihtin one year (Phragmites resource, 1989:15), we set the generation time to 2–4 years because the underground rhizome is perennial. We set the mutation rate to be 2.17e^−9^ substitutions per site per year^73^. Migration events between *P. australis* lineages were tested using Treemix^74^. All pure individuals from the lineages were included in the analysis, and SNPs were filtered to include only those present in all populations. We analyzed scenarios with 1 to 5 migration events, with each scenario tested in 5 replicates. The optimal number of migration events was determined using the R package “optM”, based on the second-order rate of change in likelihood^75^.

To determine derived alleles in *Phragmites* species, we aligned the genome reads of *O. thomaeum* (NCBI accession number: SRR2083765), *A. donax* (accession number: SRR431920), *A. plinii* (accession number: SRR4319202), *S. bicolor*(accession number: SRR25730657) and *M. sinensis* (accession number: SRR10230189) to the reference genome using bwa mem, and then obtained the consensus ancestral genome using ANGSD^76^. Ancestral alleles of the variants identified by the refmap pipeline were called and annotated using samtools and bcftools. The multidimensional unfolded SFS for the four tetraploid lineages was calculated using the easySFS program (https://github.com/isaacovercast/easySFS). The optimal projection was selected by balancing the number of segregating sites with the number of individuals. Parameters related to population splitting were estimated using fastsimcoal version 2.8^77^, and mainly focused on the divergence time between populations. Several divergence models with migration for the EU, Med, and CN lineages were assumed (see Supplementary Fig. S7 for the models tested). In each cycle, the program ran for 200,000 iterations to estimate the expected SFS and conduct 40 optimization (ECM) cycles to estimate the parameters. To find the best-fitting parameter, we performed 100 runs for each scenario, and selected the best run by the highest likelihood. The best demographic model among the three lineages was selected using AIC criteria. Based on the Stairway Plot results, all populations except USnat were considered to have constant population size, while USnat was assigned a growth rate of 0.0000066 backwards in time, indicating an exponential decrease of population sizes.

## Supplementary information

Supplementary Tables

Supplementary Information

## Data Availability

The data underlying this article are available in NCBI SRA database and can be accessed with the BioProject ID PRJNA753984.

## Code availability

The scripts used for the reads mapping, SNP calling, as well as population genetic analysis in this article could be found from github https://github.com/smallfishcui/PhragPopGen.
