# Supplementary material for: Genome-wide analysis tracks the emergence of intraspecific polyploids in Phragmites australis
Source: NPJ Biodivers. 2024 Oct 1;3:29. doi: 10.1038/s44185-024-00060-8 (PMC11445247; doi:10.1038/s44185-024-00060-8)
Supplement: Supplementary file 2 — Supplementary Information [file 44185_2024_60_MOESM2_ESM.pdf]

Supplementary materials

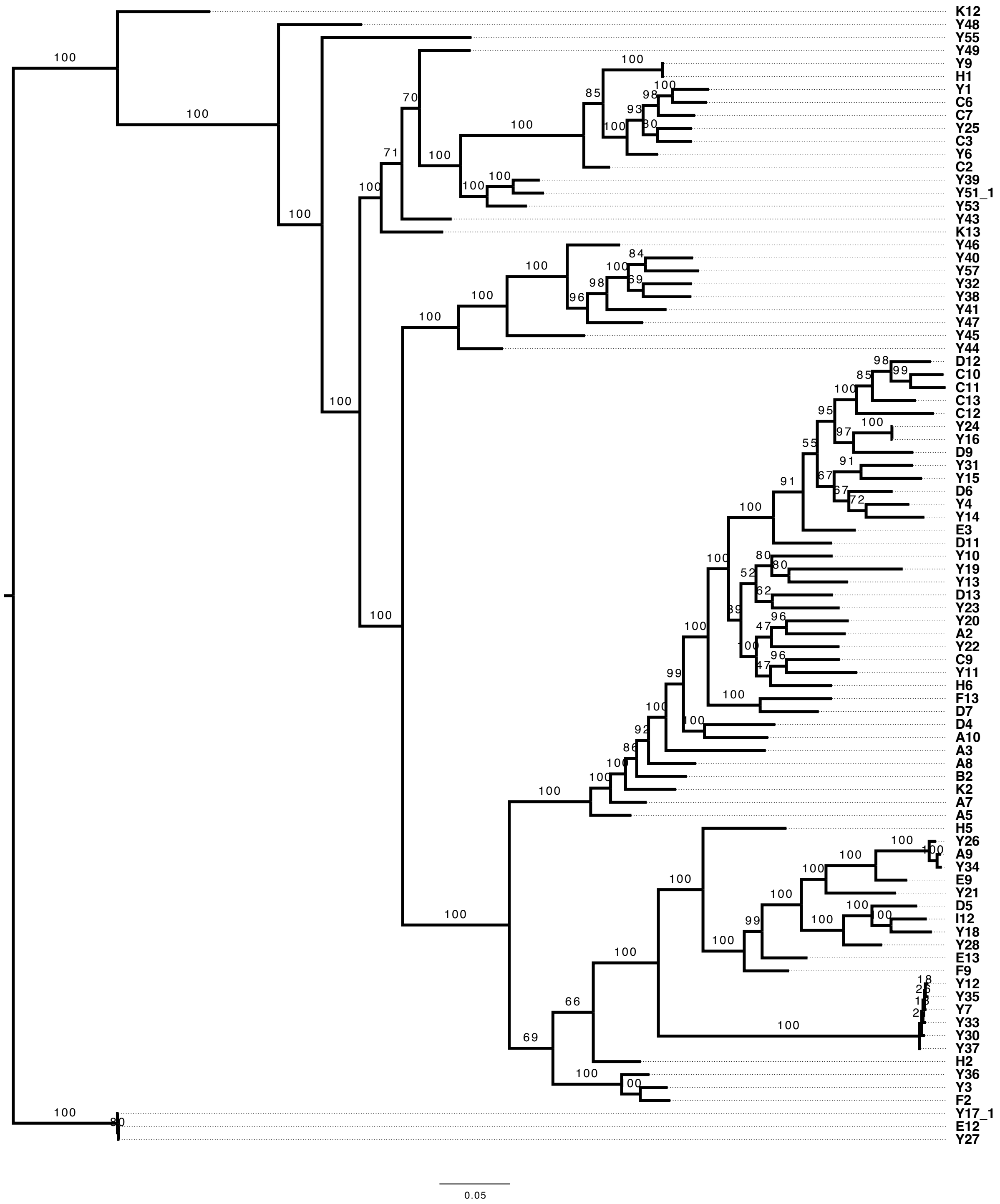

Figure S1. Nuclear phylogeny tree with bootstrap values on the branches. The tree was estimated by biallelic SNPs for 88 individuals using RAxML.

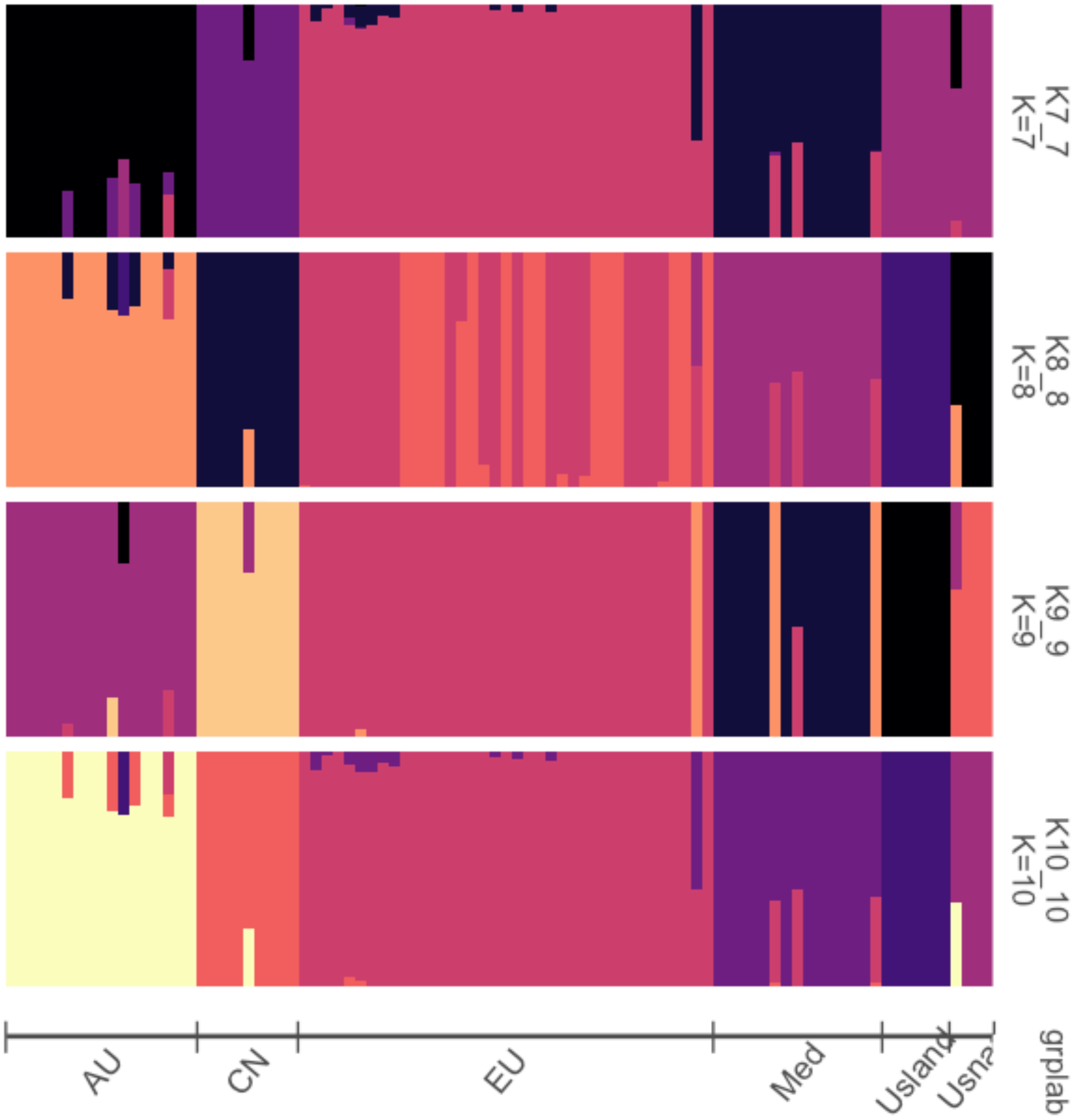

Figure S2. Genetic admixture between populations based on independent biallelic SNPs with less than 50% of missing data, SNPs with minor allele frequency less than 5% were filtered out. This resulted in a dataset of 92,769 variants, which were used in the genetic admixture analysis using ADMIXTURE. Different K values (representing the number of ancestral populations) reveal similar patterns for the admixed individuals. The best K value is 10 (of which 4 clusters were empty) as revealed by the highest likelihood.

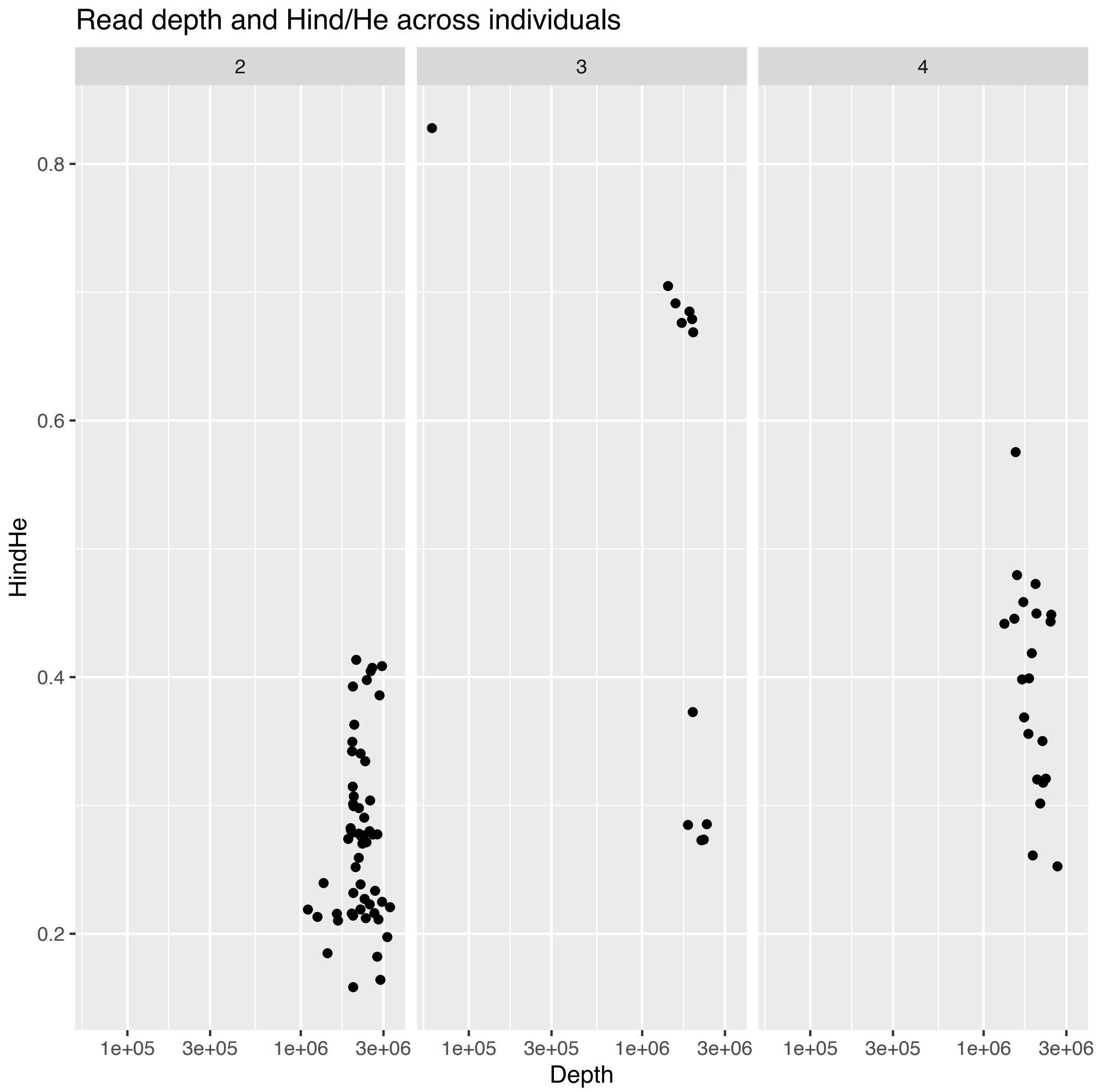

Figure S3. Hind/He value estimated using polyRAD. Hind is the probability that two sequencing reads, sampled without replacement, are different alleles (RAD tags). He is the expected heterozygosity, estimated from allele frequencies by taking account into mapping depth of RAD tags for each individual, instead of calling the genotypes. The x-axis shows the depth of reads, and the y-axis shows the Hind/He value. Each dot represents an individual. The left panel showed the Hind/He values evaluated for the allotetraploids of *P. australis*, the middle panel showed the Hind/He values evaluated for the hexaploids, and the right panel showed the evaluations for the octoploids. The highest outlier in the middle panel indicated a totally different outgroup species, *Arundo donax*; the group of outliers in the middle panel indicated the USland lineage, which resulted from hybridization of *P. australis* and *P. mauritianus*, are allohexaploids; The lower groups for the three panels from left to right indicated diploidized allotetraploids, auto-allohexaploids, and auto-allo-octoploids.

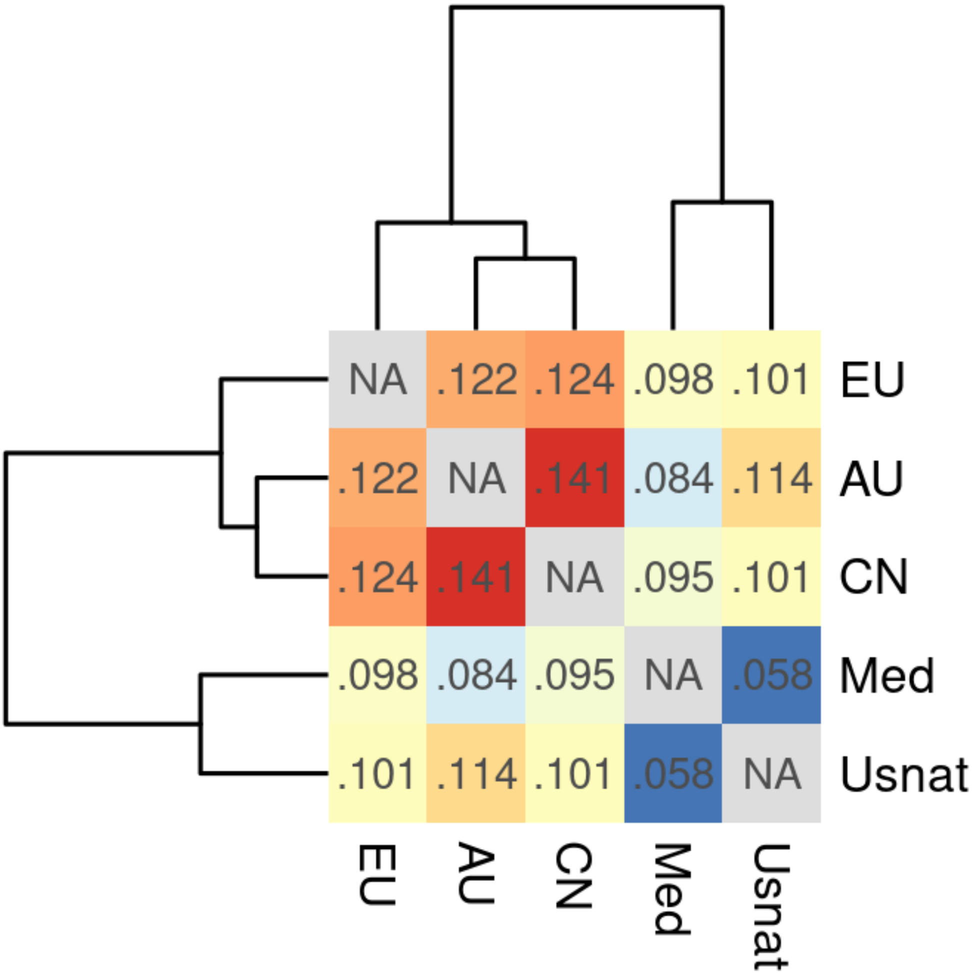

Figure S4. Heatmap showing the introgression test f3 statistics for USland lineage using admixtools 2.

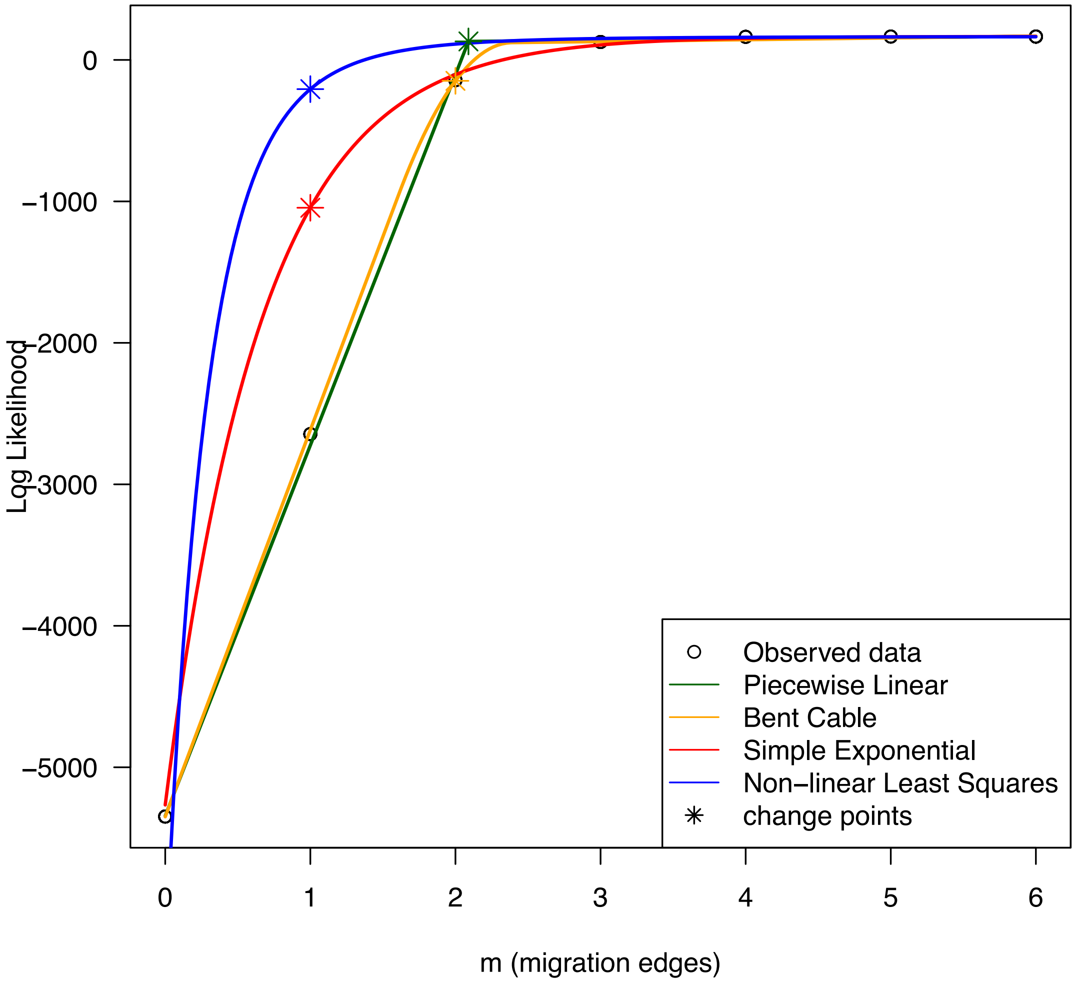

Figure S5. Parameter selection for treemix analysis. The selection of optical number of events was done using R package ‘optM’. The highest likelihood was obtained at m=2.

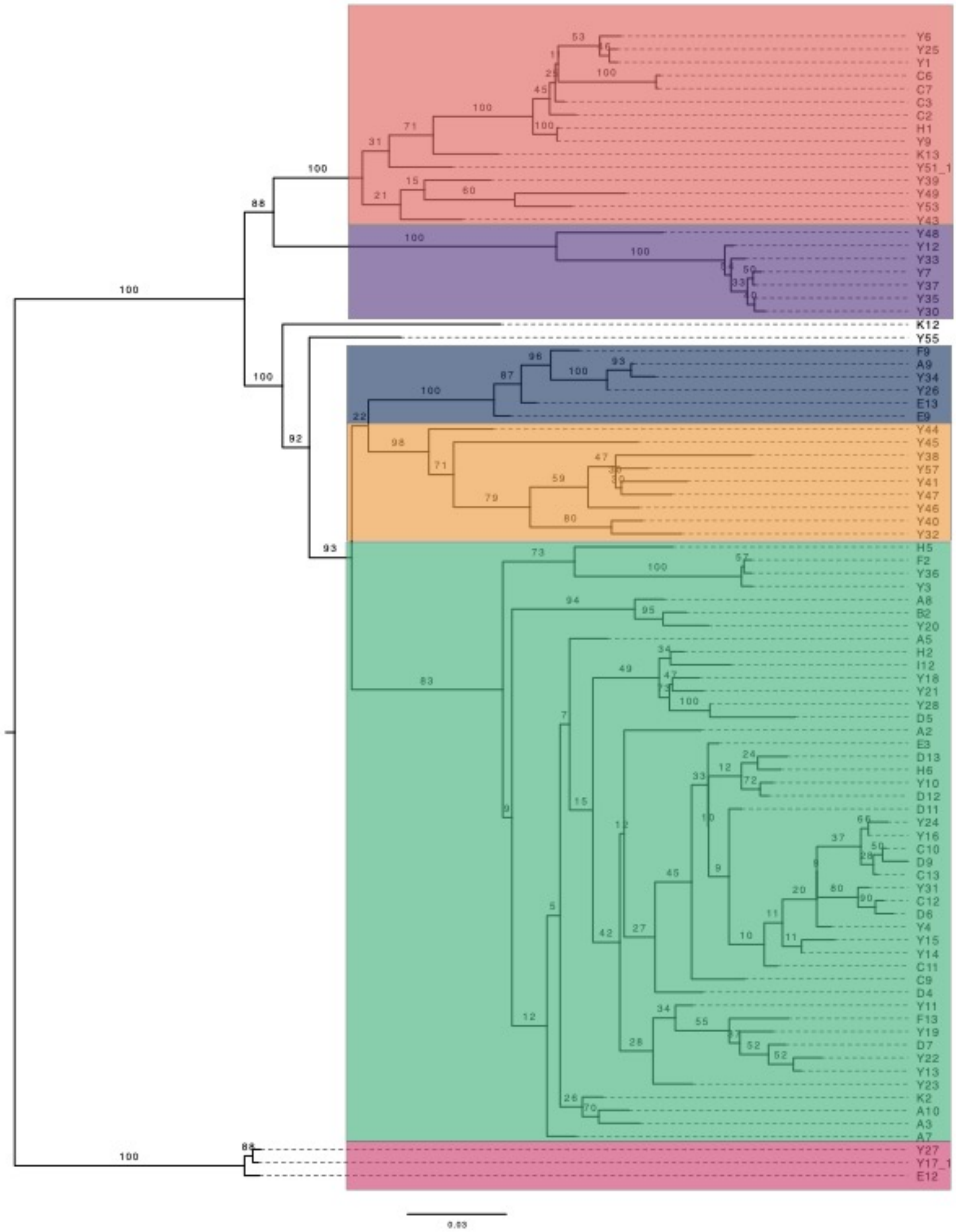

Figure S6. Rooted phylogenetic tree inferred from chloroplast SNPs using RAxML-ng. The values on the nodes give bootstrap support of the split, obtained by resampling the SNPs for 100 bootstrap runs. The color highlight indicates the genetic lineages identified by the nuclear phylogeny tree.

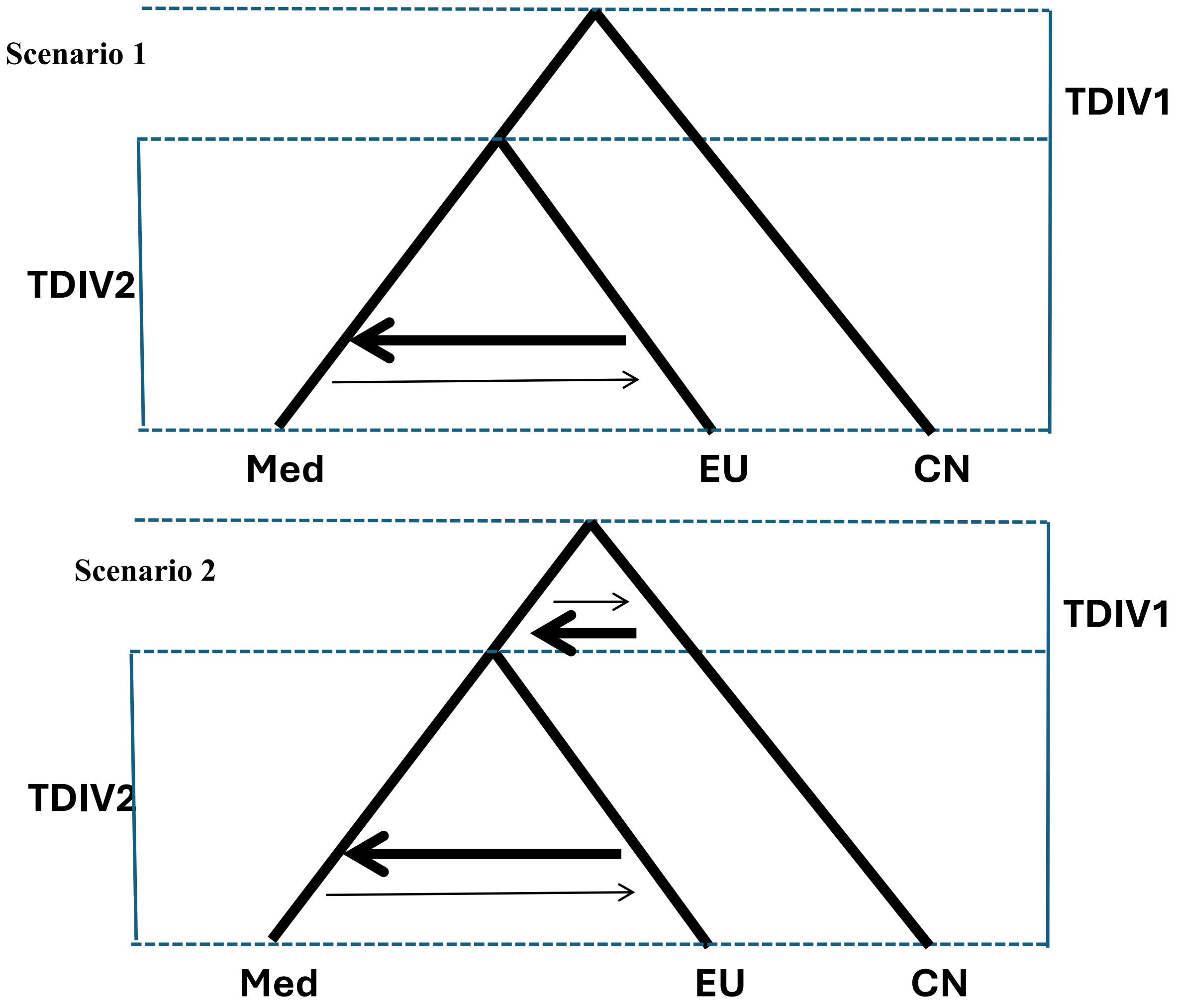
Scenario 1
TDIV1
TDIV2
Med
EU
CN
Scenario 2
TDIV1
TDIV2
Med
EU
CN

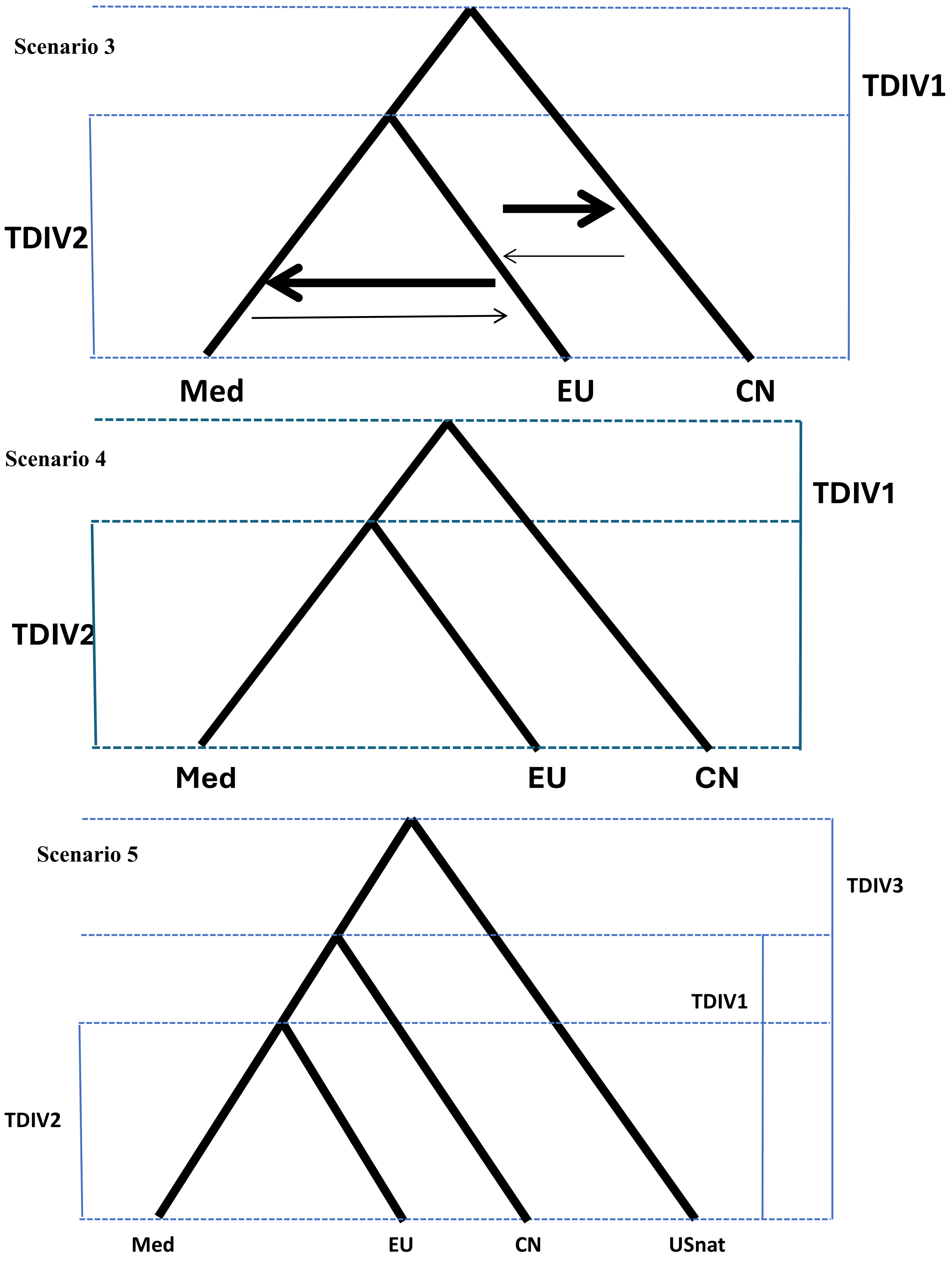

Figure S7. Demographic models simulated using fastsimcoal. Scenario 1: Constant asymmetric gene flow exists between Med and EU after the lineage divergence, no gene flow

exists between CN and other lineages. **Scenario 2:** Constant asymmetric gene flow exists between Med and EU after the lineage divergence. Constant gene flow exists between CN and the ancestor of EU and Med lineages. **Scenario3:** Constant asymmetric gene flow exists between Med and EU, CN and EU after the lineage divergence. **Scenario4:** No gene flow between any population after divergence of CN, EU and Med. **Scenario5:** No gene flow between any population after divergence of CN, EU, Med and USnat.
